# Comparing effects of intraoperative fluid and vasopressor infusion on intestinal microcirculation

**DOI:** 10.1038/s41598-020-76983-6

**Published:** 2020-11-16

**Authors:** Chia-Ning Fan, Szu-Jen Yang, Po-Yuan Shih, Ming-Jiuh Wang, Shou-Zen Fan, Jui-Chang Tsai, Wei-Zen Sun, Chih‑Min Liu, Yu-Chang Yeh

**Affiliations:** 1 Department of Anesthesiology, National Taiwan University Hospital, Chung Shan S. Rd, No.7, Taipei, 10002 Taiwan; 2 Institute of Medical Device and Imaging, College of Medicine, National Taiwan University, Taipei, Taiwan

**Keywords:** Experimental models of disease, Blood flow

## Abstract

**S**everal studies have revealed that vasopressor may be more appropriate for treating intraoperative hypotension and preventing hypervolemia. This study compared the effects of vasopressor infusion and fluid supplementation on intestinal microcirculation during treating intraoperative hypotension. Thirty-two rats were randomly divided into the following four groups: Light Anesthesia group (LA, 0.8–1% isoflurane); Deep Anesthesia group (DA, 1.5–1.8% isoflurane); Fluid DA group (1.5–1.8% isoflurane and fluid supplementation); and Norepinephrine DA group (1.5–1.8% isoflurane and norepinephrine infusion). At 240 min, perfused small vessel density (PSVD) of the mucosa did not differ significantly between the Fluid DA and Norepinephrine DA groups [26.2 (3.2) vs 28.9 (2.5) mm/mm^2^, *P* = 0.077], and tissue oxygen saturation of the mucosa was lower in the Fluid DA groups than in the Norepinephrine DA groups [ 48 (7) vs 57 (6) %, *P* = 0.02]. At 240 min, TSVD and PSVD of the seromuscular layer were higher in the Norepinephrine DA group than in the Fluid DA group. Fluid administration was higher in the Fluid DA group than in the Norepinephrine DA group [66 (25) vs. 9 (5) μL/g, *P* = 0.001]. Our results showed that norepinephrine can resuscitate intraoperative hypotension related microcirculatory alteration and avoid fluid overload.

## Introduction

**Figure 1:**
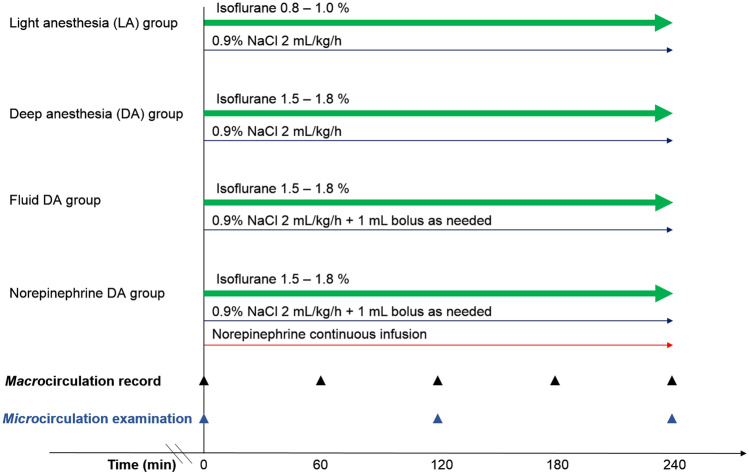
Grouping and treatment and examination protocol. The therapeutic goal of hypotension was to maintain a mean arterial pressure decreased by less than 10 mm Hg of the initial value.

Perioperative tissue hypoperfusion may result in postoperative complications and increased mortality^1^. The common causes of perioperative hypoperfusion are general anesthesia-induced hypotension, blood loss, and fluid loss. Intraoperative administration of crystalloids is commonly used to treat perioperative hypoperfusion. However, research has proved that fluid loading has no influence on anesthesia-induced hypotension^2^, and excessive fluid supplementation may cause hypervolemia-related glycocalyx impairment^3^, tissue edema, respiratory distress syndrome^4^, and abdominal compartment syndrome^5^. Postoperative fluid overload has been demonstrated to be strongly correlated with mortality^6^. Several studies have shown that vasopressors may be more appropriate for treating anesthesia-induced hypotension and prevent hypervolemia^7,8^. Furthermore, microcirculatory dysfunction can occur in the absence of arterial hypotension^9–12^. Intestinal microcirculatory dysfunction can lead to the disruption of the intestinal mucosal barrier and the development of multiple organ dysfunction syndrome^13^. To the best of our knowledge, no study has compared the treatment effects of fluid supplementation and vasopressor infusion on intestinal microcirculation. The primary aim of this study was to compare the treatment effects of fluid supplementation and vasopressor infusion on intestinal microcirculation during intraoperative anesthesia-related hypotension in an open abdominal surgery rat model (Fig. 1).

## Results

### Characteristics, treatments, and systemic hemodynamics

**Figure 2:**
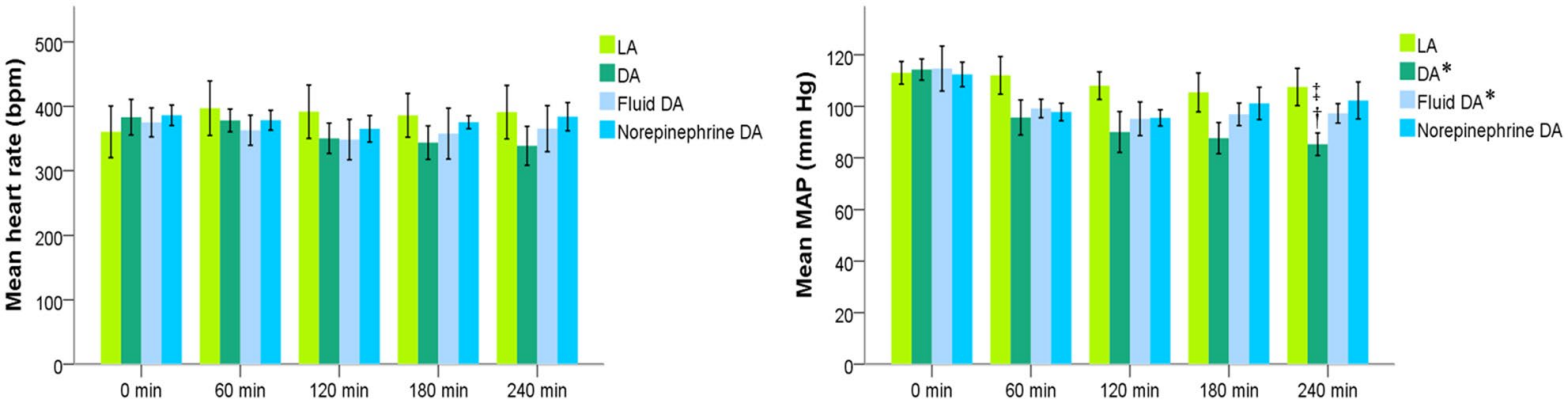
Heart rate and Mean arterial pressure (MAP) of the 4 groups. *DA* deep anesthesia, *LA* light anesthesia. *P < 0.05 vs. the LA group using repeated measures. At 240 min, ^†^*P* < 0.05 vs. the LA group and ^‡^*P* < 0.05 vs. the Norepinephrine DA group using one-way ANOVA with the Tukey test.

The mean body weights and standard deviation of the rats were 266 (8) g in the light anesthesia (LA) group, 265 (21) g in the deep anesthesia (DA) group, 253 (23) g in the Fluid DA group, and 266 (13) g in the Norepinephrine DA group. According to the treatment protocol, the mean total fluid supplementation and standard deviation for 240 min was 8 μl/g in the LA and DA groups. Fluid supplementation was higher in the Fluid DA group than in the Norepinephrine DA group [66 (25) vs. 9 (5) μL/g, *P* = 0.001]. The mean requirement and standard deviation of norepinephrine for 240 min was 40 (11) mcg in the Norepinephrine DA group. Mean arterial pressure (MAP) and the heart rate are given in Fig. 2. In the DA group, isoflurane induced 14% and 21% decreases in MAP at 60 min and 240 min, respectively, compared with the LA group. In repeated measures analysis, MAP was lower in the DA and Fluid DA groups than in the LA group. At 240 min, heart rate did not significantly differ between the Fluid DA and Norepinephrine DA groups [384 (26) vs 365 (43) beats per minute, *P* = 0.788]. At 240 min, MAP did not significantly differ between the Fluid DA and Norepinephrine DA groups [102 (9) vs 97 (5) mm Hg, *P* = 0.493].

### Intestinal microcirculation of the mucosa

**Figure 3:**
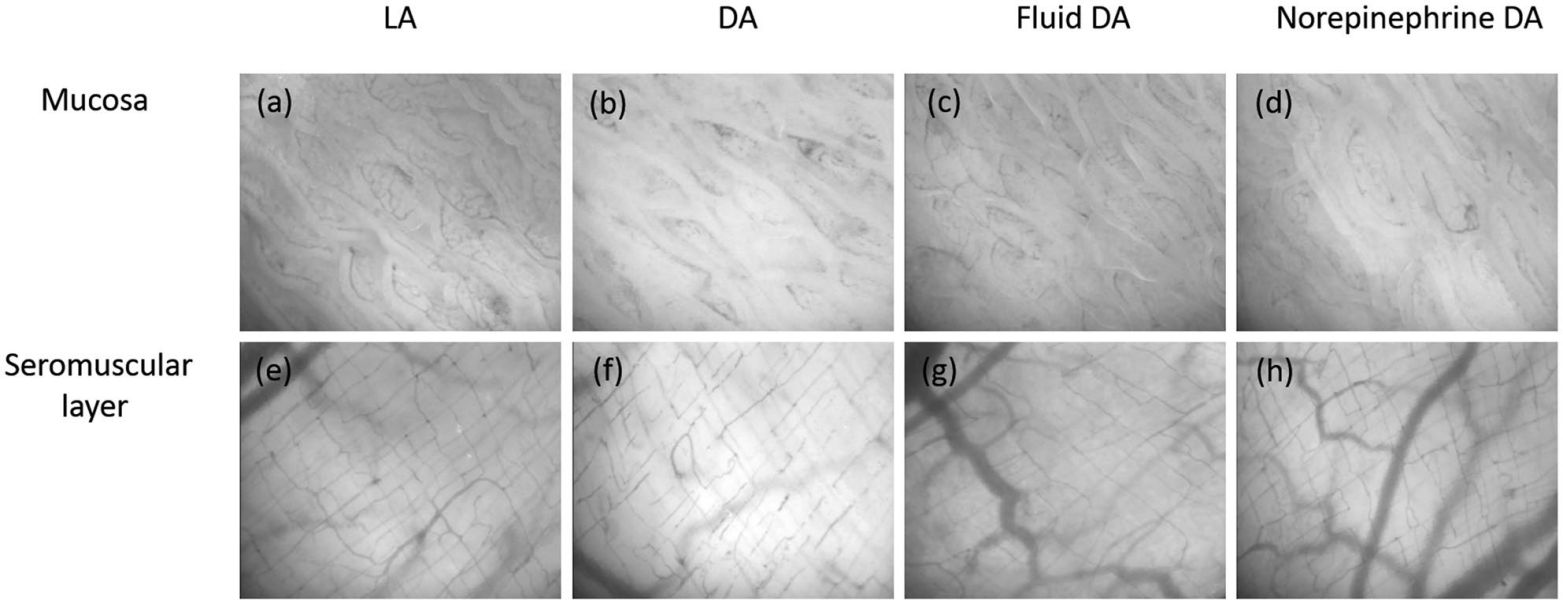
Microcirculation images of the terminal ileal mucosa and seromuscular layer. Microcirculation images of mucosa are shown in LA group (**a**), DA group (**b**), fluid DA group (**c**), and Norepinephrine DA group (**d**) at 240 min. Microcirculation images of muscular layer are shown in LA group (**e**), DA group (**f**), Fluid DA group (**g**), and Norepinephrine DA group (**h**) at 240 min. In the DA group, perfused small vessels were fewer in the intestinal mucosa (**b**) and seromuscular layer (**f**). Norepinephrine infusion could restore microcirculation in the intestinal mucosa (**d**). *DA* deep anesthesia, *LA* light anesthesia.

**Figure 4:**
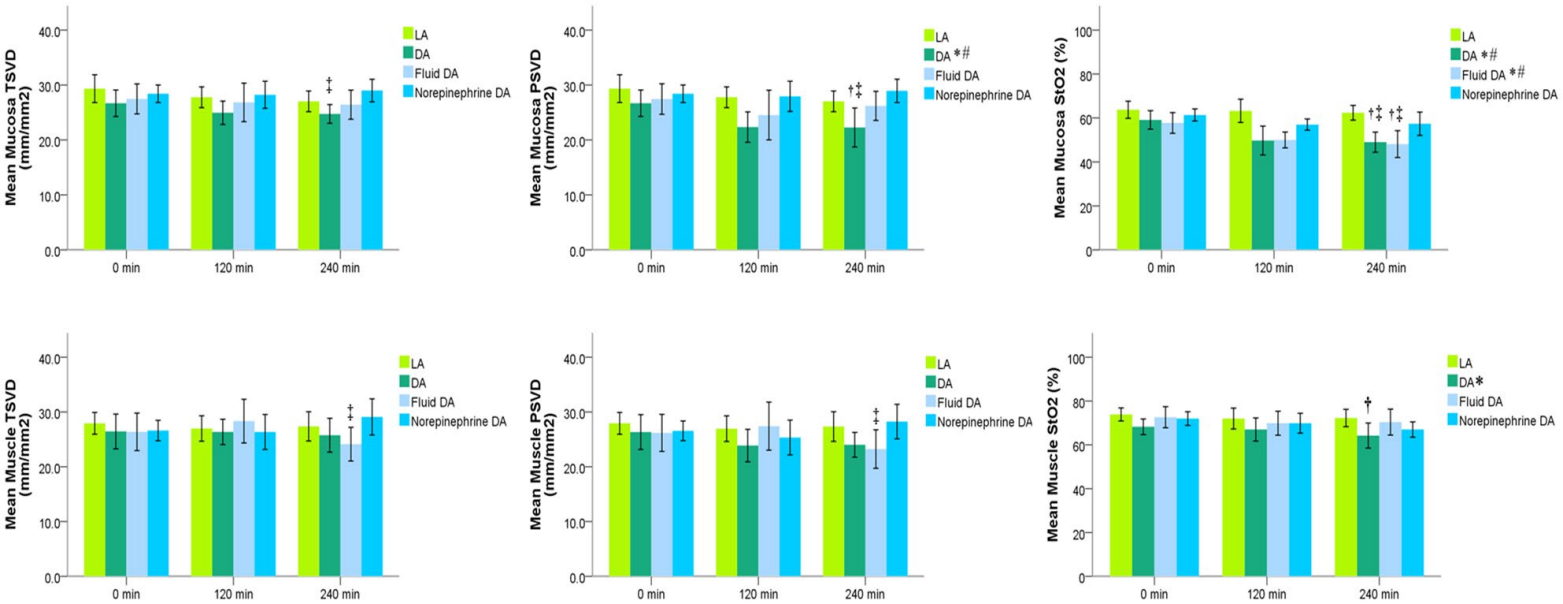
Microcirculatory parameters and tissue oxygen saturation of the terminal ileal mucosa and seromuscular layer. Primary outcome was compared using t test, and PVSD of the mucosa did not differ significantly between the Fluid DA and Norepinephrine DA groups [26.2 (3.2) vs 28.9 (2.5) mm/mm^2^, *P* = 0.077]. *DA* deep anesthesia, *LA* light anesthesia, *PSVD* perfused small vessel density, *StO*_*2*_ tissue oxygen saturation, *TSVD* total small vessel density. *P < 0.05 vs. the LA group and ^#^*P* < 0.05 vs. the Norepinephrine DA group using repeated measures. At 240 min, ^†^*P* < 0.05 vs. the LA group and ^‡^*P* < 0.05 vs. the Norepinephrine DA group using one-way ANOVA with the Tukey test.

The microcirculation images of the mucosa and seromuscular layer at baseline and 240 min are shown in Fig. 3. For primary outcome comparison at 240 min, perfused small vessel density (PVSD) of the mucosa did not differ significantly between the Fluid DA and Norepinephrine DA groups [26.2 (3.2) vs 28.9 (2.5) mm/mm^2^, *P* = 0.077]. Results of other exploratory variables are as follows. In repeated measures analysis, PSVD of the mucosa was lower in the DA group than in the LA and Norepinephrine DA groups (Fig. 4), but PSVD of the mucosa did not significantly differ between the DA and Fluid DA groups. Total small vessel density (TSVD) did not significantly differ among the four groups. At 240 min, TSVD and PSVD of the mucosa in the Norepinephrine DA group were higher than those in the DA group [TSVD 29.0 (2.5) vs 24.7 (2) mm/mm^2^, *P* = 0.011; PSVD 28.9 (2.5) vs 22.3 (4.2) mm/mm^2^, *P* = 0.001]. At 240 min, TSVD and PVSD of the mucosa did not differ significantly between the DA and Fluid DA groups [TSVD: 24.7 (2.0) vs 26.4 (3.2) mm/mm^2^, *P* = 0.545; PSVD: 22.3 (4.2) vs 26.2 (3.2) mm/mm^2^, *P* = 0.083]. At 240 min, TSVD of the mucosa did not differ significantly between the Fluid DA and Norepinephrine DA groups [26.4 (3.2) vs 29.0 (2.5) mm/mm^2^, *P* = 0.195]. In repeated measures analysis, StO_2_ of the mucosa was lower in the DA and Fluid DA groups than in the LA and Norepinephrine DA groups (Fig. 4). At 240 min, tissue oxygen saturation (StO_2_) of the mucosa was lower in the Fluid DA groups than in the Norepinephrine DA groups [48 (7) vs 57 (6) %, *P* = 0.02].

### Intestinal microcirculation of the seromuscular layer

In repeated measures analysis, TSVD and PSVD of the seromuscular layer did not differ significantly among the four groups (Fig. 4). At 240 min, TSVD and PSVD of the seromuscular layer were higher in the Norepinephrine DA group than in the Fluid DA group [TSVD: 29.1 (4.0) vs 24.1 (3.7) mm/mm^2^, *P* = 0.05; PSVD: 28.3 (3.8) vs 23.2 (4.2) mm/mm^2^, *P* = 0.039]. In repeated measures analysis, StO_2_ of the seromuscular layer was lower in the DA group than in the LA groups (Fig. 4). At 240 min, StO_2_ of the seromuscular layer was lower in the DA group than in the LA groups [72 (5) % vs 64 (7) %, *P* = 0.045], but StO_2_ of the seromuscular layer did not significantly differ among the DA, Fluid DA, and Norepinephrine DA groups.

## Discussion

Our study did not show a significant difference of PSVD of the intestinal mucosa at 240 min between norepinephrine infusion and fluid administration. From the results of comparisons of other exploratory variables, we found that norepinephrine improve tissue oxygenation of the intestinal mucosa and the microcirculation of the terminal seromuscular layer with less amount of fluid when compared with fluid administration.

Our results that norepinephrine infusion resulted in better intestinal microcirculation than fluid supplementation may indicate that norepinephrine can be safely used to restore intraoperative anesthesia-related hypotension. This finding is supported by the animal study of Hiltebrand et al.^14^, in which low to moderate doses of norepinephrine were found to preserve hemodynamic stability without any major concerns related to splanchnic organ perfusion and oxygenation. Norepinephrine actively expel blood from the splanchnic capacitance vessels, causing a rapid increase in the venous return. Pathophysiologic mechanisms of anesthesia-related hypotension include vasodilation, impairment of sympathetic nervous system, and compromised baroreflex regulation^15^. Norepinephrine can attenuate vasodilation and decrease the requirement of fluid supplement. Our results support that the adequate use of vasopressor can significantly decrease the amount of fluid administered. Over-administration of saline can lead to hyperchloremic metabolic acidosis and can damage the endothelial glycocalyx^16^. In addition, administrating a large volume of crystalloids to maintain normovolemia can reduced capillary perfusion and tissue oxygen partial pressure^17^. Aggressive fluid therapy leads to increased cardiac filling pressures, which results in the release of natriuretic peptides, and these peptides markedly disrupt the glycocalyx structure and function, leading to increased vascular leakage^18^. Moreover, the resuscitation effect of fluid supplementation may be limited or not persistent. Norberg et al. reported that a bolus of 25 mL/kg saline administered to healthy volunteers during isoflurane anesthesia had no effect on decreased blood pressure^2^.

A relevant concern is that difference in microcirculatory alterations between the mucosa and seromuscular layer. In the present study, compared with seromuscular layer, microcirculation was more severely affected in the mucosa during intraoperative hypotension. In a septic shock pig model, Hiltebrand et al. suggested that decreased capillary density may cause intestinal mucosal acidosis^19^. Mucosal microcirculatory dysfunction may account for the development of intestinal edema, stress ulcer bleeding, and bacterial translocation. Conversely, microcirculatory dysfunction was more severe in the seromuscular layer than in the mucosa during fluid supplementation for intraoperative hypotension. We suggested that overloaded fluid may result in more tissue edema in the seromuscular layer than in the mucosa. Hydrostatic edema has been reported to activate the signal transduction pathway that results in intestinal contractile dysfunction^20^. Microcirculatory dysfunction in the seromuscular layer may account for ileus.

Coherence of the macrocirculation and the microcirculation is the correlation between systemic hemodynamic variables and microcirculatory variables^21^. If hemodynamic coherence exists, correcting systemic hemodynamic variables is able to correct microcirculatory perfusion and oxygen delivery^21^. However, many studies have described conditions of a loss of hemodynamic coherence between macrocirculation and microcirculation^22,23^. We then did a post-hoc investigation of the correlation among heart rate, MAP, TSVD, PSVD, and StO_2_ at 240 min. We found that MAP was weakly correlated with PSVD (correlation coefficient = 0.363, *P* = 0.041) and StO_2_ (correlation coefficient = 0.530, *P* = 0.002). Further studies are warranted to investigate the coherence of microcirculation variables and other systemic hemodynamic variables (eg. cardiac output, urine output) or laboratory data (eg. lactate level).

This study has several limitations. First, in the Fluid DA group, the MAP goal could not be maintained even with aggressive volume resuscitation. This observation is compatible with the result of Norberg et al.^2^. Second, the anesthetized rats were under spontaneous breathing. Mechanical ventilation with considerably positive end-expiratory pressure can lead to splanchnic hypoperfusion and marked decreases in hepatic, portal venous and mesenteric blood flow^24^. Third, our model was an open abdominal surgery model. Further studies in rat model with an intact abdominal wall, the effect of increase intra-abdominal pressure resulted from excessive intestinal edema should be considered. Fourth, as our previous description of the hyperchloremic metabolic acidosis after over-administration of saline, it is warranted for further studies to investigate the treatment effect of a balanced fluid on microcirculation.

In conclusion, norepinephrine can resuscitate intraoperative hypotension related microcirculatory alteration and avoid fluid overload. Moreover, overload fluid may impair microcirculation in the intestinal seromuscular layer. We suggest that the resuscitating effects on microcirculation should be considered in further studies of investigating perioperative hypotensive resuscitation.

## Methods

### Animals

In this study, 32 male Wistar rats (body weight 250 ± 50 g; Biolasco Taiwan Co., Taipei, Taiwan) were used, and this study was approved by the Animal Care and Use Committee of the Laboratory Animal Center of the College of Medicine, National Taiwan University, Taipei, Taiwan (No. 20130287). The rats were maintained on a 12-h light–dark cycle with free access to water and food. The rats were handled according to the Guidelines for the Treatment of Laboratory Animals of the Institute Animal Care and Use Committee of National Taiwan University.

### Grouping and treatment protocol

The 32 rats were randomly assigned to four groups (8 rats in each group) (Fig. 1). In the LA group, the rats received 0.8–1% (the inspiratory concentration) isoflurane inhalation, with a continuous infusion of 0.9% sodium chloride at 2 mL/kg/h as the maintenance fluid supplementation through an external jugular vein catheter. In the DA group, the rats received 1.5–1.8% isoflurane inhalation with a continuous infusion of 0.9% sodium chloride at 2 mL/kg/h. In the Fluid DA group, rats receiving 1.5–1.8% isoflurane inhalation with a continuous infusion of 0.9% sodium chloride, and the infusion rate was adjusted to maintain MAP decreased by less than 10 mm Hg of the initial value. The rats were given a 1 mL bolus of 0.9% sodium chloride for persistent hypotension. In the Norepinephrine DA group, rats received 1.5%-1.8% isoflurane inhalation with a continuous intravenous infusion of norepinephrine (60 mcg/mL in 5% dextrose), and the infusion rate was adjusted to maintain MAP decreased by less than 10 mmHg of the initial value. Subsequently, they were administered a bolus of 1 mL of 0.9% sodium chloride for persistent hypotension. The infusion concentration of norepinephrine was determined by our pilot study.

### Anesthesia and surgical procedure

Anesthesia, tracheostomy, and right common carotid artery and external jugular vein cannulation were performed as described in our previous study^25^. Atropine sulfate (0.05 mg/kg in 0.9% sodium chloride) was administered subcutaneously to reduce respiratory tract secretions and block vagal reflexes elicited by the manipulation of the intestinal viscera. Arterial blood pressure and the heart rate were continually monitored via the right common carotid artery catheter and recorded at baseline, 60 min, 120 min, 180 min, and 240 min. The rectal temperature was continuously monitored. A 3 cm long midline laparotomy was performed to exteriorize a segment of terminal ileum which was located at 6 to 10 cm proximal to the ileocecal valve. The mucosa and seromuscular layer of the terminal ileum were prepared for microcirculation examination as described in our previous study^26^. Euthanasia was performed by exsanguination cardiac arrest under anesthesia after finishing all examinations.

### Measurement of intestinal microcirculation and tissue oxygen saturation

After a 15-min stabilization period, baseline microcirculation examinations for the mucosa and seromuscular layer was performed with a sidestream dark-field video microscope (MicroScan, Microvision Medical, Amsterdam, The Netherlands) and a tissue oxygen monitor (white light reflectance spectroscopy, moorVMS-OXY, Moor Instruments Ltd., Devon, UK). The time after the examination was set as 0 min, and the rats received treatment according to their grouping. Intestinal microcirculation was examined again at 120 min and 240 min.

Intestinal microcirculation parameters, including TSVD (less than 20 μm) and PSVD were analyzed using semi-automated analysis software (AVA 3.0, Academic Medical Center, University of Amsterdam, Amsterdam, Netherlands) according to the guidelines of microcirculation analysis^27,28^. At each time point, three continuous image sequences (10 s) were digitally stored for each measured site, and the data of the three images were averaged for obtaining the required statistics. Analyses were performed by a single investigator who was blinded to grouping. At each time point, StO_2_ was measured at three points of the intestinal mucosa and seromuscular layer, and the data of these points were averaged for statistical calculation.

### Primary outcome and sample size calculation

Primary outcome in this study was defined as the difference of PSVD of the mucosa between the Fluid DA and Norepinephrine DA group. Based on our experience^26,29^, this study (n = 8 rats per group) was powered to detect a 10% difference of PSVD of the mucosa between the two groups at 240 min, with an α level of 0.05 (single-tailed) and a β level of 0.2 (80% power), assuming a control PSVD of 29 (2.3) mm/mm^2^.

### Statistical analysis

Data analysis was performed using statistical software (SPSS 20; IBM SPSS, USA). Data normality was examined by W/S test^30^, and appropriate statistical analysis was used according to the normality of data. Primary outcome was examined by the Mann–Whitney U-test or the t-test according to the normality of PSVD. A *P* value of < 0.05 was considered significant. Other exploratory variables were examined as follows. Differences in means over time among the four groups were analyzed using general linear model repeated measures ANOVA (Analysis of Variance). Differences in means at 240 min among the four groups were analyzed using one-way ANOVA, followed by the Tukey test for post hoc analysis. The *P* values for comparisons of exploratory data were not adjusted.

## Data Availability

The datasets generated during and/or analysed during the current study are available from the corresponding author on reasonable request.
